# COVID-19 and Ecosyndemic Vulnerability: Implications for El Niño-Sensitive Countries in Latin America

**DOI:** 10.1007/s13753-020-00318-2

**Published:** 2020-11-13

**Authors:** Ivan J. Ramírez, Jieun Lee

**Affiliations:** 1 Department of Health and Behavioral Sciences, University of Colorado Denver, Denver, CO 80217 USA; 2 Consortium for Capacity Building, Institute of Arctic and Alpine Research (INSTAAR), University of Colorado Boulder, Boulder, CO 80308 USA; 3 Department of Geography, GIS, and Sustainability, University of Northern Colorado, Greeley, CO 80639 USA

**Keywords:** Coronavirus, COVID-19, Ecosyndemic, El Niño, El Niño-Southern oscillation, Infectious disease vulnerability, Latin America

## Abstract

Latin America has emerged as an epicenter of the COVID-19 pandemic. Brazil, Peru, and Ecuador report some of the highest COVID-19 rates of incidence and deaths in the region. These countries also face synergistic threats from multiple infectious diseases (that is, ecosyndemic) and quasi-periodic El Niño-related hazards every few years. For example, Peru, which is highly sensitive to El Niño, already copes with an ecosyndemic health burden that heightens during and following weather and climate extreme events. Using an ecosyndemic lens, which draws on a multi-disease hazard context of place, this commentary highlights the importance of El Niño as a major factor that not only may aggravate COVID-19 incidence in the future, but also the broader health problem of ecosyndemic vulnerability in Latin America.

## Introduction

This commentary highlights the importance of El Niño-Southern Oscillation (ENSO) for infectious disease vulnerability in Latin America amidst the ongoing novel coronavirus (COVID-19) pandemic. It builds on previous work in the region focused on climate, ENSO, and health within a multi-disease hazard context of place (Confalonieri et al. 2009; Ramírez et al. 2018; Ramírez 2019), influenced by Singer’s (2009) concept of “ecosyndemic.” Like a multi-hazard approach to disasters (UNDRR 2015), the notion of ecosyndemic conceptualizes hazards, in this case infectious diseases, from a broader lens that examines an epidemic in relation to rather than independent of other epidemics. As Ramírez et al. (2018) describe, ecosyndemics are public health phenomena where simulataneous disease hazards overlap interconnected by environmental changes and social vulnerabilities. From this lens, the COVID-19 pandemic is a broader public health hazard that may be exarcerbated by climate variability and extreme events, namely El Niño, the warm phase of ENSO, which threatens Latin America every few years.

The pandemic, which first emerged in late 2019, is a global health emergency, infecting approximately 28.6 million people, and killing 917,000 persons, as of 13 September 2020 (WHO 2020). Like other disasters, not every country is equally vulnerable. An analysis by the World Bank concluded that “with more people living close to the international poverty line in the developing world, low- and middle-income countries will suffer the greatest consequences [of the virus] in terms of extreme poverty” (Mahler et al. 2020). The United Nations assessments (UN 2020a, b) suggest that impacts of COVID-19 affect the most vulnerable countries, many already facing multiple human development challenges, including inadequate access to clean water, clean air, nutrition, sanitation, and shelter. Furthermore, populations in these countries also face increasing societal exposure and vulnerability to all hazards and subsequent adverse health outcomes, including epidemics.

**Fig. 1:**
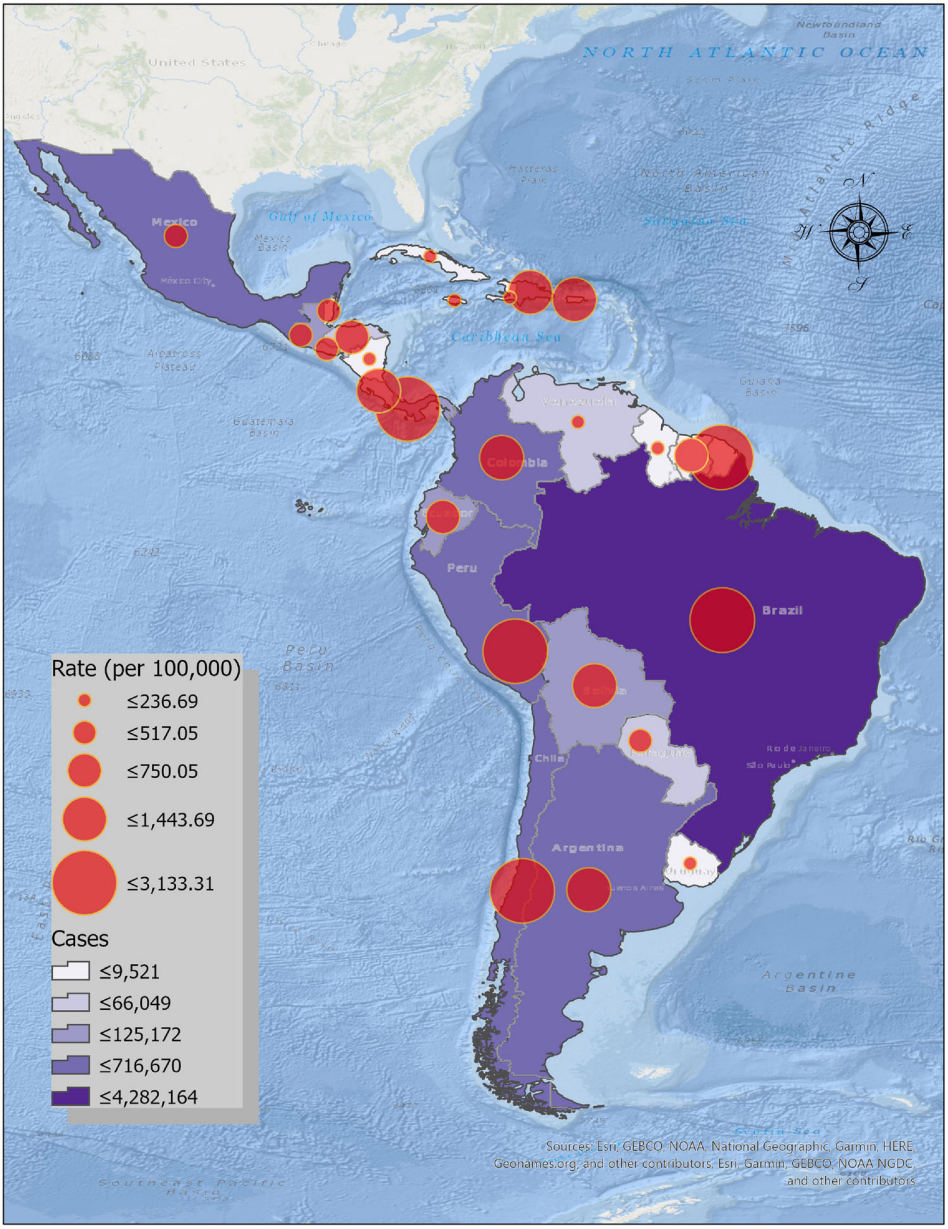
COVID-19 cases and incidence rates (per 100,000 persons) in Latin America and the Caribbean, as of 13 September 2020. *Data source* WHO (2020), U.S. Census Bureau (2020)

The Latin American region is an epicenter for COVID-19 (PAHO 2020a; UN 2020b), representing approximately one-third of all cases and deaths worldwide (WHO 2020). Figure 1 shows COVID-19 incidence rates (per 100,000 persons) across Latin America and the Caribbean, as of mid-September 2020. In Brazil, Peru, Colombia, Mexico, and Chile alone (of which Brazil shared the greatest burden), the total number of cases was approximately 7.3 million (WHO 2020). The proportions of people diagnosed with COVID-19 who died (case fatality ratio) have also been increasing across the region. Table 1 indicates that countries with some of the highest case fatality ratios were also countries with the highest incidence rates, including several of the Andean countries, mentioned earlier. Moreover, PAHO (2020a) estimated that one in four persons in the region would experience severe health outcomes, due to the growing prevalence of chronic underlying preconditions, such as diabetes and cancer.

**Table 1:** Ten countries in Latin America and the Caribbean by highest COVID-19 case fatality ratio (confirmed deaths per 100 cases) with incidence rates (confirmed cases per 100,000 persons), 13 September 2020. *Data source* WHO (2020), U.S. Census Bureau (2020)

Country	Incidence rate (per 100,000)	Case fatality ratio (%)
Mexico	517.05	10.66
Ecuador	686.88	9.44
Bolivia	1090.95	5.79
Peru	2266.21	4.25
Nicaragua	63.10	3.71
Guatemala	484.12	3.61
Colombia	1443.69	3.21
Honduras	724.28	3.12
Brazil	2036.20	3.05
Guyana	236.69	2.95

## The Ecosyndemic Context

Of urgent concern is how COVID-19 might compound preexisting infectious disease vulnerability in the region (Burki 2020; Rodriguez-Morales et al. 2020). Countries in Latin America and the Caribbean are already burdened with multiple infectious disease hazards (Schneider et al. 2011; PAHO 2020b), many preventable, such as dengue, malaria, and leishmaniasis. Table 2 displays the ecosyndemic potential of countries with a high number of vector-borne diseases, including several arboviruses. In 2016, some countries reported as many as nine (Ecuador) to 11 (Brazil) vector-borne diseases, as well as high rates of diarrheal and respiratory-related infections. Such an array of disease hazards not only reflects societal vulnerability—such as disproportionate exposure of communities of lower socioeconomic status, and inadequate infrastructure (McCormick and Lang 2016; Engels and Zhou 2020), but also highlights the region’s climate suitability for infectious diseases (Confalonieri et al. 2009; Campbell-Lendrum et al. 2015), which raises concerns about COVID-19 and the threat of El Niño, given its association with and impact on a number of disease hazards in the region (Stewart-Ibarra and Lowe 2013; Ramírez and Grady 2016; Caminade et al. 2017).

**Table 2:** Ecosyndemic potential of countries with high number of vector-borne diseases in Latin America. *Data source* PAHO (2020b)

Vector-borne disease	Brazil	Ecuador	Peru	Guatemala
Chagas	x	x	x	x
Chikungunya	x	x	x	x
Dengue	x	x	x	x
Leishmaniasis	x	x	x	x
Lymphatic Filariasis	x			
Malaria	x	x	x	x
Onchocerciasis	x	x		x
Plague	x	x	x	
Schistosomiasis	x			
Yellow Fever	x	x	x	
Zika	x	x	x	x
Total	11	9	8	7

## The El Niño Factor

The El Niño phenomenon, which stems from ocean–atmosphere interactions across the equatorial Pacific Ocean, affects regional to local weather patterns every few years (McPhaden et al. 2006). El Niño is often associated with water, weather, and climate-related extremes and changes in seasonality (Naranjo et al. 2018), that in turn influence local disease ecologies and population exposures (Kovats et al. 2003; McGregor and Ebi 2018; Anyamba et al. 2019). El Niño’s impact on disease transmission occurs directly via ecological changes (for example, hydrology and rising ambient and water temperatures), which may propogate a variety of pathogens, as well as spawn a variety of hydrometeorological (hydromet) hazards, including floods, temperature extremes, windstorms, and droughts. During El Niño’s onset, several months of anomalous temperatures, both ambient and coastal water, may precede rainfall anomalies (dry and wet, depending on the geography), which in turn precipitate floods or droughts that impact disease hazards (for example, in the case of cholera, see Jutla et al. (2013) or Ramírez et al. (2016)). Indirectly, El Niño-related hazards are also sources of health vulnerability (Ebi and Bowen 2015), through physical impacts on the built environment and infrastructure (for example, damaging or overwhelming water and sanitation systems), and long-term stresses (for example, societal impacts on livelihoods, population displacement) that may follow post-El Niño years (Glantz 2001a, 2001b; Ramírez 2019). Among countries in Latin America, some of the most affected by COVID-19 are also vulnerable to the effects of El Niño on their societies and health. For example, countries in South America such as Peru, Ecuador, and Brazil are highly sensitive to El Niño-related hydromet hazards and subsequent disasters (Caviedes 1984; Cornejo and Zavala 2017; Marengo et al. 2018), including infectious disease epidemics (Confalonieri et al. 2009; Stewart-Ibarra and Lowe 2013; Sorensen et al. 2017).

Often, El Niño’s impacts aggravate preexisting health burdens, and heighten social vulnerabilities (for example, compromising potable water access, sanitation, hygiene, nutrition, and/or livelihoods) to hydromet and other hazards. Thus, it is a convergence and cascade of hazards and disasters that often heighten health vulnerability (Thomas et al. 2020). One example is the case of the zika virus and 2016 earthquake in Ecuador. As Sorensen et al. (2017) describe, anomalous ambient temperatures spawned favorable environmental conditions for zika-carrying mosquitoes during an El Niño that preceded a catastrophic earthquake. Subsequently, the number of zika cases surged, although correlation is not always causation. Another example is epidemic cholera in Peru in 1991. Preceding the emergence of cholera were several disasters and social hardships in the prior year that included an earthquake (northeast Peru), a climate-induced agricultural disaster (highlands), energy crisis (northern Peru), and stringent economic restructuring policies (Fujishock) to address hyperinflation (Ramírez et al. 2013). Following the onset of the epidemic in January 1991 was the development of a moderate El Niño, contributing to torrential rains and flooding in early 1992, increasing vulnerability to cholera in the austral summer. In the context of COVID-19, Thomas et al. (2020) presents examples from Kenya and Puerto Rico, and states, “dealing with COVID-19 response and other natural disasters [sic] poses significant challenges in resources and balancing approaches […] Some might consider this compounding events, but given the stresses on social, economic, and political systems that never have an opportunity to recover, the cascade may occur in downward spiraling social vulnerability” (Thomas et al. 2020, p. 7).

## El Niño and Ecosyndemic Vulnerability: The Case of Peru

**Fig. 2:**
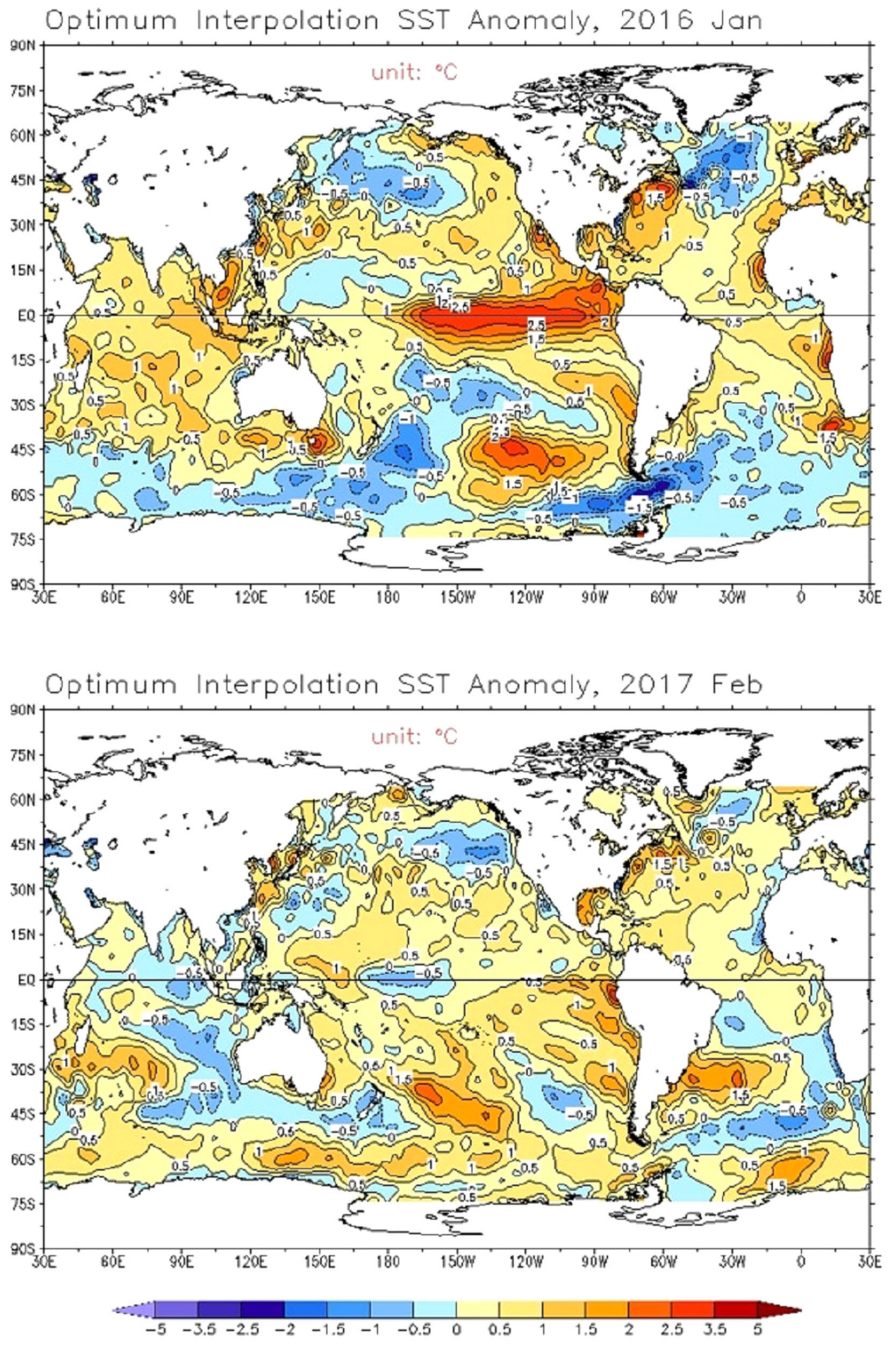
Sea surface temperature anomalies (°C, from red to blue indicating above average to below average) across the equatorial Pacific Ocean for January 2016 (top panel) and February 2017 (bottom panel). *Data source* https://www.cpc.ncep.noaa.gov/products/GODAS/monthly.shtml

Peru, historically vulnerable to El Niño, provides a case example to explore ecosyndemic vulnerability as a warning context for COVID-19. For example, during the 1982–1983 and 1997–1998 El Niños, the two strongest of the twentieth century, Peru reported anomalous temperatures, catastrophic flood-related disasters, and simultaneous disease outbreaks, including rises in malaria, cholera (1998 only), pneumonia, conjunctivitis, and diarrheal diseases (non-cholera) (Gueri 1984; Hijar et al. 2016; Ramírez 2019). More recently, the country endured two successive El Niños, including the 2014–2016 event, and a localized coastal event (Costero) in 2017 (Rodriguez-Morata et al. 2018), the latter episode being the most severe. Figure 2 illustrates the intensity of sea surface temperature anomalies for the 2014–2016 and 2017 El Niños. Compared to the 2014–2016 episode, the 2017 El Niño Costero was acute in its onset, limited in its spatial extent (Peru and Ecuador), and came without early warning to the Andean region (Ramírez and Briones 2017).

According to PAHO, several epidemics associated with arboviruses such as dengue, chikungunya, and zika, as well as zoonotic infections like leptospirosis emerged in Peru following simultaneous flooding events (Ministry of Health Peru 2015a; PAHO 2017; Silva Chávez and Hernández Córdova 2017). Table 3 displays case counts for several infectious diseases and related groups of infections (for example, diarrheal) from 2014 to 2017 in Peru. As the table indicates, several diseases increased following the onsets of the two recent El Niño events (November 2014 and January 2017): dengue, diarrheal, and respiratory in 2015 and diarrheal, leptosporisis, pneumonia, and respiratory infection (non-pneumonia) in 2016. Of particular impact on public health was dengue and leptosporisis, diseases with three and six times higher prevalence in 2017 compared to the previous year.

**Table 3:** Selected infectious diseases case counts in Peru from 2014 to 2017 during two successive El Niños *Data source* Ministry of Health, Peru (2015b, 2016, 2017, 2018a, b, 2019a, b)

Infectious disease	2014	2015	2016	2017
Dengue	17,234	35,816	25,159	76,093
Diarrheal	1,010,745	1,118,569	1,194,505	1,181,169
Leishmaniosis	6849	5999	7458	6023
Leptospirosis	34	25	88	545
Malaria	65,258	63,192	56,533	55,227
Pneumonia	25,896	25,158	26,405	26,112
Respiratory	2,582,330	2,626,857	2,793,147	2,595,308

What is troubling from this case example is not only the burden of disease, but that the epidemics tend to cluster in place and time during and in the aftermath of El Niños and environmental changes. For example, Ramírez et al. (2018) investigated population vulnerability to seven infectious diseases (diarrheal disease (non-cholera), cholera, two types of malaria, conjunctivitis, respiratory infection (non-pneumonia), and pneumonia) in Piura of northern Peru during the 1997–1998 El Niño. Using a spatial index approach, the authors showed that patterns of ecosyndemic risk intensified over time across several districts and varied spatially as the extreme events (for example, heavy rains) progressed. Ramírez et al. (2018) also showed that ecosyndemic disease burden was correlated with urban density in low-lying areas largely affected by flooding (for example, number of people affected as a proxy for disaster impact) in Piura. In another example, Tallman et al. (2020) examined the ecosyndemic relationships between human activities like dam construction and roads with an ensemble of vector-borne and sexually-transmitted diseases in Brazil and Peru. In this study, the findings suggest that ecosyndemics varied by place, but risk overall was explained by a complex intersection of environment changes and psychosocial stressors.

## COVID-19 as an Additional Health Burden

**Fig. 3:**
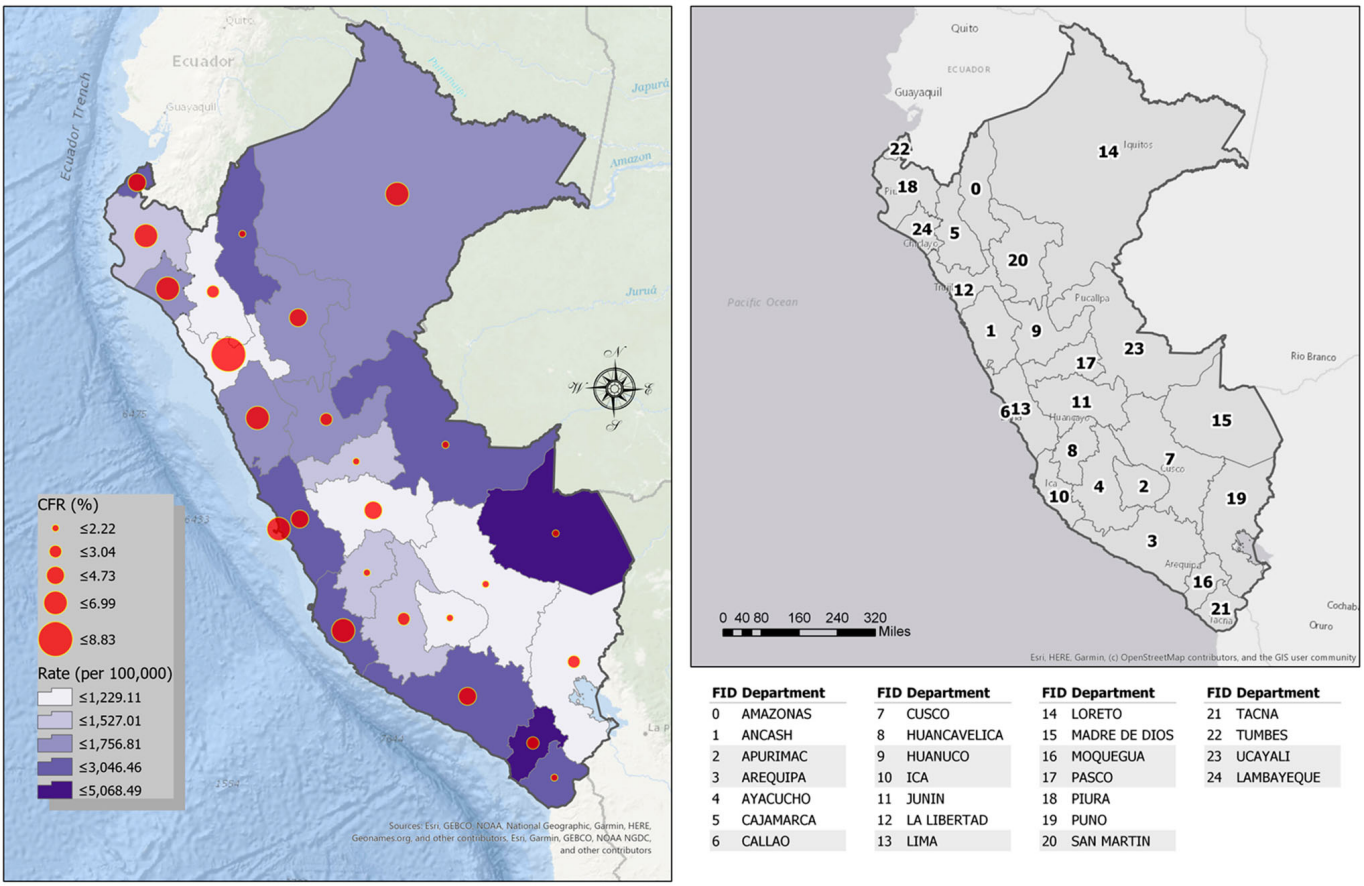
COVID-19 incidence rate (per 100,000 persons) and case fatality ratios (CFR, %) in Peru, as of 5 September 2020. A reference map is also shown with the feature identification (FID) for the geographical unit (department). *Data source* Ministry of Health, Peru (2020a)

For El Niño-affected countries like Peru, COVID-19 presents an additional health burden to manage within a broader context of ecosyndemic vulnerability. Figure 3 shows COVID-19 rates of incidence and case fatality ratios in Peru at the department level, as of 5 September 2020. According to the map, the distribution of COVID-19 in Peru was widespread. Initially when Latin America emerged as a hotspot, the highest incidence rates and case fatality ratios were mainly located along the arid low-lying coast in the north as well as jungle regions (for example, Loreto) in the east (see Table 4 for a comparison of departments with the highest incidence rates and case fatalities for 29 May and 5 September). By 5 September, the spatial distribution of incidence shifted to the central and southern parts of Peru. During this time (May to September), however, the greatest proportion of people dying from COVID-19 remained in the central and northern coast and two jungle regions. Among the departments, Piura and Tumbes, which border Ecuador in the north, stand out as areas well known for El Niño-related health and disaster impacts (Bayer et al. 2014; Ramírez 2019; French et al. 2020). Table 5 shows that Piura and Tumbes reported the most damage during the recent El Niño Costero event along with five other departments (Lambayeque, Ica, Ancash, Loreto, and La Libertad) where case fatality ratios for COVID-19 were also the highest. The point here is that underlying conditions in these areas create social vulnerability to multiple hazards, which persist over years, and are further complicated by concurrent hazard issues like those influenced by El Niños.

**Table 4:** Comparison of highest incidence rates and case fatality ratios by department in Peru on 29 May and 5 September 2020 *Data source* Ministry of Health, Peru (2019c, 2020a, 2020b)

Incidence rate (per 100,000)		Case fatality ratio (%)	
29 May	5 September	29 May	5 September
**Callao**	Moquegua	**Lambayeque**	**La Libertad**
**Lima**	**Madre de Dios**	**Ica**	**Lambayeque**
**Ucayali**	**Lima**	**Ancash**	**Ica**
Lambayeque	Amazonas	**Loreto**	**Ancash**
**Tumbes**	Tacna	**Piura**	**Piura**
Loreto	**Callao**	**Tumbes**	**Loreto**
Piura	**Ucayali**	**La Libertad**	**Callao**
Ancash	**Tumbes**	Amazonas	**Tumbes**
**Ica**	Arequipa	Ucayali	San Martin
**Madre de Dios**	**Ica**	**Callao**	Junin

Bold indicates departments where incidence rates and case fatality ratios remained in the top 10

**Table 5:** Departments with most populations affected by the 2017 El Niño Costero *Data source* INDECI (2017), Ministry of Health, Peru (2019c)

Department	Population affected (per 100,000)
**Piura**	18,419.9
**Tumbes**	17,950.5
**Lambayeque**	11,600.3
**Ica**	11,258.1
**Ancash**	9518.2
**Loreto**	9242.0
Huacavelica	8275.8
**La Libertad**	7423.8
Madre de Dios	5350.5
Arequipa	3479.6

Bold indicates departments where COVID-19 case fatalities have been highest in 2020

While it is unknown precisely how El Niño will affect COVID-19 incidence in Peru in the future, it is foreseeable that hydromet hazards may exacerbate transmission and ecosyndemic vulnerability in certain geographic regions, although this may vary with the strength and dynamics of a particular El Niño, and especially changing social and health conditions of local communities, governance, and health system preparedness. El Niño-related hydromet hazards and seasonal changes may influence local ecologies of COVID-19 and other endemic disease hazards (for example, diarrheal diseases and dengue) directly and indirectly, as described earlier. Impacts on disease may be mediated through local temperature and rainfall extremes and subsequent hydromet hazards, which vary by geography (for example, floods in northern Peru, drought in Peruvian Amazon). Furthermore, hydromet-related disasters may compromise the public health response to COVID-19 outbreaks. For example, during the 2017 El Niño Costero event, an estimated 934 health posts were badly damaged due to flooding (French et al. 2020). As of July 2020, only 4.0% of 150 health stations scheduled for reconstruction were rebuilt (Zapata et al. 2020). Thus, a cascade of hydromet-related hazards may heighten COVID-19 and ecosyndemic risks across the country.

## Concluding Thoughts

Coincidently, as COVID-19 emerged globally, so was the onset of a weak El Niño—see NOAA (2020) for the Oceanic Niño Index, which inspired this preliminary work. This commentary highlights the importance of El Niño not to suggest that climate triggered the COVID-19 pandemic, but rather to place the emergence within a broader context of climate and ecosyndemic vulnerability.

In many cases, the novel COVID-19 is now one of many infectious disease hazards co-circulating in socially vulnerable countries that face socioeconomic and infrastructure challenges. For example, the percentage of people in urban areas living in slum-like conditions with several basic needs unmet was 20.9 in Latin America and the Caribbean, based on 2018 estimates (UN 2020c). Such conditions may limit the ability of populations to take prevention measures, such as adequate hygiene practice (for example, frequently washing hands with soap), social distancing, and wearing face masks (UN 2020b). According to UNICEF (2020), the region reports that 39.0% of populations have limited handwashing facilities or do not have such facilities with soap and water. In El Niño-affected countries like Peru, at least 26.0% of the healthcare facilities do not have hand hygiene facilities, while Ecuador reports 3 million people without basic handwashing facilities in homes. In Brazil, 35.0% of schools do not have hygiene facilities for handwashing (UNICEF 2020). Furthermore, many people work in informal sectors of the economy (for example, 71% in Peru), and therefore, cannot minimize their exposure like those with the ability to stay at or work from home (Enriquez et al. 2020).

Country-level health system capacities to cope with and respond to COVID-19 are also limited in the region. As Litewka and Heitman (2020) point out, health systems in Latin America are underfunded to begin with, and many countries’ budgets for healthcare are only 4.0% of the gross domestic product, limiting access to quality healthcare, resources, and personnel to fight the pandemic. Taking into account the pandemic and ecosyndemic context, health systems can become overburdened. As one researcher states, “Brazil has an excellent public health system, but it cannot cope with competing crises…and could easily end up in a situation where there is a surge of all vector-borne diseases” (as quoted in Burki 2020).

Although it was initially argued that climate (for example, seasonality) could possibly constrain the spread of COVID-19 (for example, Sajadi et al. 2020), the emergence of Latin America, particularly South America, as an epicenter of COVID-19, suggests that equatorial regions are also at great risk, as some preliminary research shows (O’Reilly et al. 2020), particularly in places with limited health systems (Merow and Urban 2020). Thus, examination of COVID-19 within a broader scope of ecosyndemic vulnerability including quasi-periodic El Niño-related threats may provide new insights for prevention and disaster risk reduction strategies that address COVID-19 and the broader public health problem of multi-infectious disease vulnerability in the region.
